# Toward G-Quadruplex-Based Anticancer Agents: Biophysical and Biological Studies of Novel AS1411 Derivatives

**DOI:** 10.3390/ijms21207781

**Published:** 2020-10-21

**Authors:** Anna M. Ogloblina, Nunzia Iaccarino, Domenica Capasso, Sonia Di Gaetano, Emanuele U. Garzarella, Nina G. Dolinnaya, Marianna G. Yakubovskaya, Bruno Pagano, Jussara Amato, Antonio Randazzo

**Affiliations:** 1 N.N. Blokhin National Medical Research Center of Oncology, Ministry of Health, 115478 Moscow, Russia; ann.ogloblina@gmail.com (A.M.O.); mgyakubovskaya@mail.ru (M.G.Y.); 2 Department of Pharmacy, University of Naples Federico II, Via D. Montesano 49, 80131 Naples, Italy; nunzia.iaccarino@unina.it (N.I.); emanueleugo.garzarella@unina.it (E.U.G.); antonio.randazzo@unina.it (A.R.); 3 Center for Life Sciences and Technologies (CESTEV), University of Naples Federico II, Via A. De Amicis 95, 80145 Naples, Italy; domenica.capasso@unina.it; 4 Institute of Biostructures and Bioimaging, National Research Council, Via Mezzocannone 16, 80134 Naples, Italy; digaetan@unina.it; 5 Department of Chemistry, Lomonosov Moscow State University, 119991 Moscow, Russia; dolinnaya@hotmail.com

**Keywords:** guanine-rich oligonucleotides, G-quadruplex, in vitro biological assays, biophysical characterization, nucleolin, antiproliferative activity

## Abstract

Certain G-quadruplex forming guanine-rich oligonucleotides (GROs), including AS1411, are endowed with cancer-selective antiproliferative activity. They are known to bind to nucleolin protein, resulting in the inhibition of nucleolin-mediated phenomena. However, multiple nucleolin-independent biological effects of GROs have also been reported, allowing them to be considered promising candidates for multi-targeted cancer therapy. Herein, with the aim of optimizing AS1411 structural features to find GROs with improved anticancer properties, we have studied a small library of AS1411 derivatives differing in the sequence length and base composition. The AS1411 derivatives were characterized by using circular dichroism and nuclear magnetic resonance spectroscopies and then investigated for their enzymatic resistance in serum and nuclear extract, as well as for their ability to bind nucleolin, inhibit topoisomerase I, and affect the viability of MCF-7 human breast adenocarcinoma cells. All derivatives showed higher thermal stability and inhibitory effect against topoisomerase I than AS1411. In addition, most of them showed an improved antiproliferative activity on MCF-7 cells compared to AS1411 despite a weaker binding to nucleolin. Our results support the hypothesis that the antiproliferative properties of GROs are due to multi-targeted effects.

## 1. Introduction

The increased understanding of tumor biology has led to the development of targeted therapies, whose benefits are documented in clinical trials and are acknowledged in their approval and licensing [1]. Targeted drugs, which are more effective and safer compared to genotoxic cancer therapeutics, provide new treatment opportunities for patients and allow us to move towards personalized therapeutic approaches. In the last years, there has been growing interest in the use of oligonucleotides as specific inhibitors of various oncoproteins for anticancer targeted therapy [2,3]. In particular, guanine-rich oligonucleotides (GROs) have drawn considerable attention thanks to the strong cancer-selective antiproliferative activity reported for many of them [4]. This is considered to arise in large part from the binding to target proteins involved in carcinogenesis and tumor progression, which occurs thanks to the peculiar three-dimensional structural motif adopted by GROs. Indeed, they can form G-quadruplexes (G4s), characteristic secondary structures composed of planar arrangements of four guanine (G) bases stabilized by eight Hoogsteen hydrogen bonds known as G-tetrads [5]. Interestingly, G4s are characterized by high thermodynamic stability, good resistance to numerous serum nucleases, increased cellular uptake, and ease of chemical synthesis and modification that make them an interesting alternative to antibodies and small molecules used in targeted therapy of cancer [6]. The pleiotropic properties of G4-forming GROs and their intrinsic high specificity against cancer cells, which is a general feature of most of the GROs studied so far, allowed to consider them as promising candidates for multi-targeted cancer therapy [4,7].

AS1411—an unmodified 26-mer G-rich oligodeoxyribonucleotide with the sequence 5′-GGTGGTGGTGGTTGTGGTGGTGGTGG-3′—represents the first-in-class anticancer GRO with a promising biological activity [8]. It has reached phase II clinical trials for acute myeloid leukemia and renal cell carcinoma [9]. However, despite a good tolerance and safety profile, the trial was terminated due to suboptimal pharmacokinetics (rapid clearance from the blood) and relatively low potency [10]. The potential clinical impact of AS1411 is also hampered by the lack of a well-defined structure. Indeed, it is well established that AS1411 forms G4 structures [10]. However, the high degree of structural polymorphism in solution makes the identification of the biologically relevant structure(s) of this GRO still an unresolved issue [10]. Trent and collaborators detected at least eight different forms of AS1411 by a size exclusion chromatography method [11]. In order to reduce the conformational polymorphism of AS1411, Phan et al. analyzed several related oligonucleotides with single nucleotide substitutions. This allowed the identification of a new AS1411-derived sequence, named AT11, able to form a single major G4 conformation in solution, i.e., a four G-tetrad-layer G4 structure composed of two propeller-type parallel-stranded subunits connected through a central linker, as determined by NMR studies [12]. Interestingly, AT11 showed an antiproliferative activity similar to AS1411 on a human lung cancer cell line, as well as no effects on the normal cell lines.

So far, different mechanisms of action have been proposed to contribute to the biological activity of GROs, most of which involving interaction with nucleolin, a multifunctional protein overexpressed in the cytoplasm and on the cell surface of many tumor types [9]. Experimental evidence indicates that AS1411 binding to nucleolin leads to dysfunction or mislocalization of numerous nucleolin-containing complexes, such as inhibition of nuclear factor-κB activation, S-phase cell cycle arrest, and reduction of bcl-2 expression, just to cite a few [8]. However, multiple nucleolin-independent biological effects have also been described so far [10,13]. Indeed, recent data reveal that AS1411 is also able to recognize STAT3 and topoisomerase I proteins and that the inhibition of topoisomerase I is a general property of G4-forming oligonucleotides [7]. Furthermore, evidence has been provided that the antiproliferative activity of GROs may also be due to the cytotoxicity of their guanine-based degradation products, i.e., deoxyguanosine monophosphate, deoxyguanosine, and guanine [14].

Another distinctive feature of G4-forming oligonucleotides is their strongly enhanced cellular uptake compared to the non-G4 sequences [15]. The cellular uptake of GROs was initially considered to be mediated only by cell surface nucleolin (as receptor), then different mechanisms such as macropinocytosis (a form of endocytosis prevalent in cancer cells) have also been proposed.

It is clear that G4-forming oligonucleotides have distinct biological properties that make them promising anticancer agents. Besides the limitation on the clinical application of AS1411, its tumor-selective properties have been extensively explored to create drug release systems for therapeutic agents and imaging probes [10], while little has been done to improve AS1411′s bioactivity. In this frame, we have designed, synthesized, and investigated a small library of AS1411 derivatives differing in the sequence length and base composition (number of G and thymine (T) residues), with the aim of assessing whether these changes can lead to bioactive GROs with improved/different properties. With this purpose, a number of biophysical techniques have been employed for studying the structure and stability of the synthesized GROs (i.e., circular dichroism and nuclear magnetic resonance spectroscopies), as well as for evaluating their ability to interact in vitro with nucleolin (by surface plasmon resonance) and to inhibit topoisomerase I. Furthermore, the resistance of such GROs to degradation by both extracellular and nuclear nucleases was analyzed by means of gel electrophoresis and fluorescence experiments. Finally, GROs cytotoxic activity against breast adenocarcinoma MCF-7 cells was analyzed by MTT assay, and apoptosis activation was measured using annexin V/PI double staining assay.

## 2. Results

### 2.1. Design and Synthesis of GROs

With the aim to find AS1411 analogs with enhanced properties and to investigate the relationships between antiproliferative activity, nucleolin binding affinity, and G4 structures, we synthesized a small library of GROs (the sequences are shown in Table 1). These sequences were designed starting from the AS1411 sequence, by varying length, loop sequence, and G content. First, we introduced one additional G for each run of contiguous G residues, thus obtaining the hereafter called AS1411-G sequence, which in principle should form a G4 with three G-tetrad layers. Then, we added to this sequence an additional T residue to each of the six single-nucleotide loops (excluding the central ‘TTGT’ loop) to obtain the 40-mer AS1411-GT sequence. In addition, we prepared two analogs of the latter GRO: AS1411-GT-T8, in which the central ‘TTGT’ loop was replaced by a longer one consisting of eight T residues, and AS1411-GT-5′tr, which is a 5′-end truncation of AS1411-GT and AS1411-GT-T8 sequences consisting of only the first nineteen residues. In particular, loops were modified since they are known to play a key role in the overall folding and stability of G4 structures [16]. Moreover, recent studies have shown that loop length is a determinant factor for nucleolin binding to G4s, independently of the G4 conformation and oligonucleotide/loop sequence [17,18].

All GROs were prepared by solid-phase oligonucleotide synthesis, using phosphoramidite chemistry, on a commercially available controlled-pore-glass (CPG) support, using 1- or 5-µmol scale synthesis, and purified by high-performance liquid chromatography (HPLC) to ensure high purity. G4s were prepared in two different phosphate-buffered solutions (pH 7.4): 10 mM Li_3_PO_4_, 10 mM KCl, 0.2 mM EDTA (hereafter referred to as K^+^ buffer), and 10 mM Na_2_HPO_4_, 1.8 mM KH_2_PO_4_, 137 mM NaCl, 2.7 mM KCl (hereafter referred to as PBS buffer). The structure and stability of GROs under these experimental conditions were then analyzed.

### 2.2. Nuclear Magnetic Resonance Analysis of GROs

The G4 structure of each GRO was first investigated by nuclear magnetic resonance (NMR) spectroscopy to evaluate similarities/differences between AS1411 and its derivatives. NMR spectroscopy is widely employed for studying GRO secondary structures [19,20]. The imino protons of G-tetrad forming guanines give rise to characteristic chemical shifts around 10.5–12 ppm [19,21]. This chemical shift region is fully separated from imino chemical shifts of any other DNA conformation, such as duplex or other secondary DNA structures, or single-stranded DNA. Thus, the imino-proton region of Gs provides a direct and clear monitoring system for the formation of a G4 structure as well as the presence of multiple G4 conformations [19,21]. Indeed, from the number of imino peaks and their line width, it is possible to determine whether there are multi-conformational species in solution and their relative populations.

**Figure 1:**
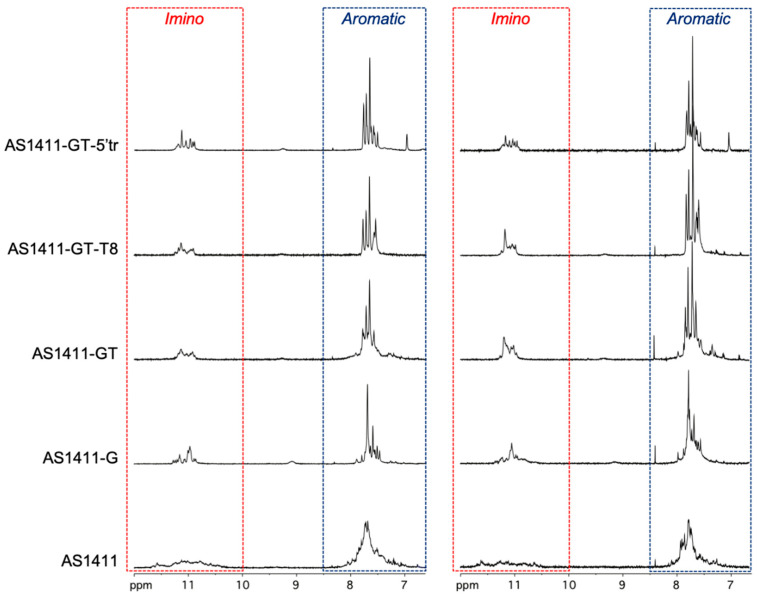
Imino and aromatic proton regions of AS1411 and its derivatives in K^+^ (**left** panel) and PBS (**right** panel) buffers.

The imino and aromatic proton regions of the 1D ^1^H NMR spectra of AS1411 and its derivatives in K^+^ and PBS buffers are shown in Figure 1. NMR spectra show only minor differences (mainly related to the number and line width of signals) between the two buffers, suggesting similar G4 foldings for all the investigated GROs in the two experimental conditions. Trent and coworkers previously demonstrated that AS1411 forms a mixture of G4 species in solution [11]. The NMR spectra clearly confirm the inherent structural polymorphism of this sequence, indeed both the imino and aromatic proton regions exhibit the presence of numerous proton signals all characterized by a broad line width (Figure 1). The absence of well-resolved proton signals in the NMR spectrum of AS1411 may also be explained by the intense exchange of imino protons with water, which occurs even at temperatures below the melting temperature of the oligonucleotide, indicating the relatively low stability of two-layer G4s formed by this GRO. On the other hand, in AS1411-G, the presence of an additional G residue for each G-tract, which in principle should promote the formation of a G4 with three G-tetrad layers, effectively decreased the complexity of the spectrum, favoring the formation of two major G4 populations as suggested by the presence of two sets of signals both in the imino and aromatic region of the spectrum. Unfortunately, signal broadening and spectral overlap hinder a detailed structural analysis and do not allow us to draw a conclusion about the G4 structural features. The further introduction of an additional T residue to each single-nucleotide loop of AS1411-G, led to a poorly resolved NMR spectrum of the corresponding AS1411-GT sequence, suggesting the presence of a heterogeneous mixture of G4 structures.

Then, the effect of the length and base composition of the central linker on the G4 structural polymorphism was also evaluated. The 1D ^1^H NMR spectrum of AS1411-GT-T8 turned out to be very similar to that of AS1411-GT, suggesting no significant effects of that linker on the conformational variability of the investigated GROs. On the other hand, the structural variability observed for AS1411-GT and AS1411-GT-T8 is not present in the case of AS1411-GT-5′tr. Indeed, the 1D ^1^H NMR spectrum of the truncated AS1411-GT-5′tr sequence showed more resolved imino signals with reduced resonance overlap, thus suggesting the formation of a single G4 population. Altogether, these results indicate that the G4 structural complexity strongly depends on the number of G-tracts in the sequence. In fact, the presence of eight G-tracts in AS1411, AS1411-G, AS1411-GT, and AS1411-GT-T8 gives in principle the possibility of forming (i) two tandem G4s connected by a linker, or (ii) bimolecular G4 structure(s), or (iii) a mixture of them.

### 2.3. Circular Dichroism Characterization of GROs

The overall G4 structures adopted by the investigated GROs in K^+^ as well as PBS buffers were further evaluated by circular dichroism (CD) spectroscopy. Independently from the buffer, the CD spectra of the five GROs all showed the typical CD signature of parallel G4 topologies, which is characterized by a positive band at around 260 nm and a negative one at around 240 nm (Figure S1) [22,23]. However, the CD spectra cannot provide indications about the presence of one or more different G4 species in a mixture, unless they are of different topologies (parallel and antiparallel). On the other hand, the thermal denaturation of G4s can be followed by the changes in the CD signals at appropriate wavelengths to provide useful information on their stability and molecularity [24,25,26]. In the case of intramolecular G4s, generally, the unfolding is a simple and fast unimolecular process that leads to true equilibrium curves, which can be used in a quantitative analysis to determine the melting temperature and other thermodynamic parameters of the DNA structures [24,25,27]. In the case of intermolecular G4s, as a result of the slow kinetics of association and dissociation of the complexes, the process is not at thermodynamic equilibrium. For these systems, the apparent melting temperatures (*T*_1/2_) are concentration-dependent and should also be influenced by the heating rate [24,28]. Therefore, recording CD melting experiments at different DNA concentrations can represent a good approach to distinguish between intramolecular and intermolecular G4s [29]. CD melting experiments were performed both in K^+^ and PBS buffers (Figure S2) by varying both the DNA concentration (1 and 20 μM single-stranded DNA) and heating rate (0.5 and 2 °C/min). Sigmoidal melting profiles were obtained in all cases, demonstrating that these GROs form stable G4 structures in both buffers. However, the GROs showed higher melting temperature values in K^+^ rather than in PBS (Table 2), in accordance with the better ability of the K^+^ ion to stabilize the G-tetrads compared to Na^+^ [30]. With the sole exception of AS1411-GT-5′tr, the melting profiles of all investigated GROs were influenced by the DNA concentration and/or heating rate, thus suggesting once again that these sequences form a mixture of G4s in solution, probably including bimolecular structures. On the other hand, the melting temperature of AS1411-GT-5′tr was not significantly affected by the sample concentration or scan rate, thus confirming the NMR data which suggests that it forms an intramolecular G4 structure.

Among the GROs, AS1411 and AS1411-G turned out to form the less and the most stable structures of the series, respectively. As far as AS1411 is concerned, the lower thermal stability could be due to the presence of only two guanines for each G-tract, which probably allows the formation of a lower number of stacked G-tetrads compared to the other investigated GROs having three guanine residues for each G-tract. On the other hand, the concomitant presence of three guanines for each G-tract and of single thymine loops in AS1411-G (which should force the formation of double-chain reversal loops) should allow the formation of more energetically-favored, and thus more stable, G4 structures [31].

### 2.4. Surface Plasmon Resonance Studies of Interaction between GROs and Nucleolin

The recognition of nucleolin on the surface of tumor cells is one of the key factors for a specific targeting and an efficient cellular uptake of AS1411 [9]. Most of the techniques used to study DNA-protein interactions provide valuable information about the affinity of interactions, with little or no insight into the kinetic parameters. However, DNA-protein interactions are dynamic, and the association and dissociation rates may affect many biological processes, such as the ability of the targeted protein to interact with other biological partners or to move between cellular compartments. Therefore, to gain a complete and biologically relevant understanding of these interactions, it is important to study them as dynamic processes. In this frame, surface plasmon resonance (SPR) represents a powerful tool to study DNA–protein interactions, since it provides both equilibrium and kinetic information [32,33]. Indeed, SPR measures the kinetic rate constants of association (*k*_on_) and dissociation (*k*_off_), leading to the estimation of the equilibrium binding constant [34].

**Figure 2:**
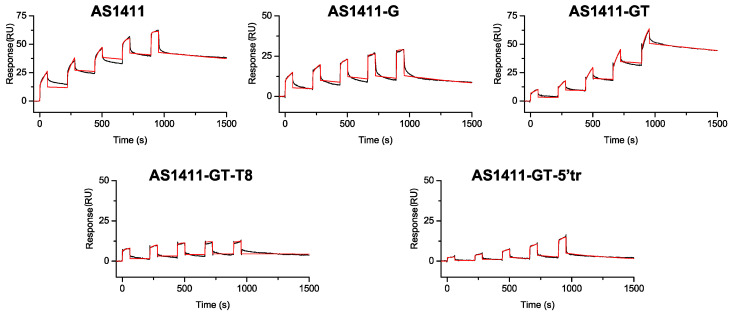
Time evolution SPR sensorgrams obtained at 25 °C by injections of various concentrations (from 2 to 32 μM) of each investigated GROs on the chip-immobilized nucleolin with a contact time of 60 s and a flow rate of 30 μL/min. The sensorgrams are shown as black lines and their respective fits as red lines.

Therefore, a biophysical analysis of the interaction of the investigated GROs with nucleolin was carried out by SPR experiments. Figure 2 shows SPR sensorgrams obtained by injecting increasing concentrations of GROs over the nucleolin-functionalized sensor-chip surface by using the single-cycle kinetics (SCK) method. SCK was preferred to the multi-cycle kinetics because, using this method, the regeneration step (which is detrimental to the protein) is performed only at the end of the complete binding cycle, thus allowing the chip surface and immobilized protein to last longer [35,36]. The GROs (analytes) were injected from a low to high concentration with short dissociation times in between and a long dissociation time at the end. After each injection of GROs, a response proportional to the concentration of injected analyte solution was observed in almost all sensorgrams (Figure 2), indicating the specificity of DNA/protein interactions [35]. Furthermore, binding responses were in the range of the maximal expected binding level (Rmax) to the protein surface, indicating a stoichiometric binding behavior. Thus, sensorgrams were fitted to 1:1 model (BiaEvaluation software) to afford the kinetic rate constants, *k*_on_ and *k*_off_, while the equilibrium dissociation constants (*K*_D_) were calculated as a ratio between *k*_off_ and *k*_on_ (Table 3).

On the basis of the results obtained by SPR, GROs’ affinity towards nucleolin can be ranked as follows: AS1411-GT-T8 >> AS1411 > AS1411-G > AS1411-GT >> AS1411-GT-5′tr. Interestingly, this rank is in agreement with the literature data, which indicate that nucleolin preferentially binds long-looped G4 nucleic acids independently of their topology [17,18]. Indeed, AS1411-GT-T8 (with a central loop of eight nucleotides) showed a *K*_D_ of 12 nM, which is around 7-fold lower than that found for AS1411 (*K*_D_ = 86 nM). Conversely, AS1411-GT-5′tr (without long loops) turned out to be the GRO with the lowest affinity of the series (*K*_D_ = 2800 nM, around 32-fold higher than AS1411), thus suggesting, once again, the importance of long loops in the GRO/nucleolin interaction. On the other hand, an intermediate affinity was observed for AS1411-G and AS1411-GT oligonucleotides having the same four-nucleotide central loop of AS1411, with *K*_D_ values of around 2- and 6-fold higher than AS1411, respectively.

### 2.5. Topoisomerase Inhibition Assay

Topoisomerase I (TOP1) is a vital enzyme that relaxes supercoiled DNA and consequently mediates chromatin dynamics, transcription, replication, DNA damage repair, and genomic stability [37]. TOP1 inhibitors have gained considerable clinical significance due to their efficacy as anticancer agents [38,39]. Recently, it has been shown that TOP1 can recognize and bind certain G4s [40], thus resulting in loss of its enzymatic activity [41,42].

Therefore, the inhibitory ability of GROs against TOP1 was investigated by analysis of electrophoregrams of the relaxation products of the supercoiled plasmid pUC19 under the action of the enzyme. The method for determining the activity of TOP1 is based on different mobility of supercoiled and relaxed forms of plasmid DNA in an agarose gel under the influence of an electric field. Supercoiled plasmid DNA has a more compact shape and its electrophoretic mobility is significantly higher than that of relaxed or nicked forms. The activity of TOP1 can be estimated by the ratio of the relaxed and supercoiled forms of plasmid DNA on an electrophoregram (Figure S3).

**Figure 3:**
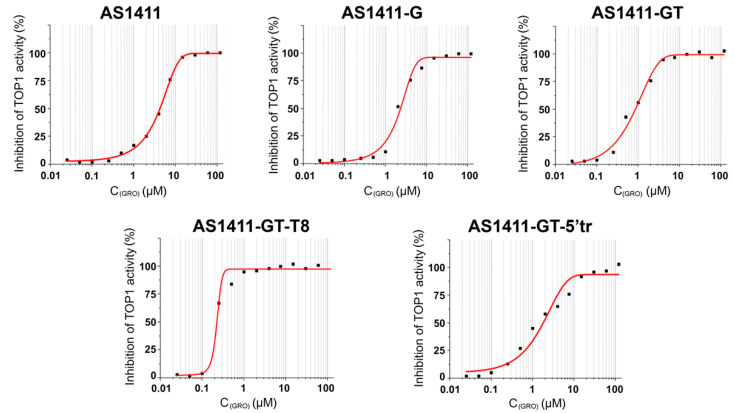
Inhibition of TOP1-catalyzed relaxation of supercoiled pUC19 plasmid in the presence of GROs. Dependence of enzyme inhibition percentage on the logarithm of GRO concentration was built according to the gel electrophoresis data quantified by ImageJ software.

As a result of TOP1 inhibition, the amount of supercoiled DNA increases as the concentration of GROs in the reaction mixture increases, while the fraction of the relaxed plasmid decreases. The degree of inhibition of the enzyme was determined as the ratio of supercoiled plasmid to the total amount of plasmid. For each concentration of GRO, the degree of inhibition of TOP1 was estimated, then the dependence of the degree of inhibition on the logarithm of the concentration of oligonucleotide was calculated (Figure 3), which was used to determine the IC_50_ value for each GRO (Table 4). Interestingly, all the investigated GROs exhibited a significant inhibitory effect against TOP1, with IC_50_ values in nano- to micromolar range. AS1411 showed the lowest TOP1 inhibition activity of the series, while the strongest inhibitor was AS1411-GT-T8.

### 2.6. Evaluation of Oligonucleotide Stability to Serum and Nuclear Extract Nucleases

Although the antiproliferative activity of GROs has been associated with various protein targets, some experimental evidence indicates that it might also be due to the cytotoxicity of guanine-based degradation products [14]. From this perspective, a good GRO candidate should exhibit great resistance to serum nucleases to reach intact the target cell, while it could be hydrolyzed in the presence of cellular nucleases to release the cytotoxic guanine-based compounds.

**Figure 4:**
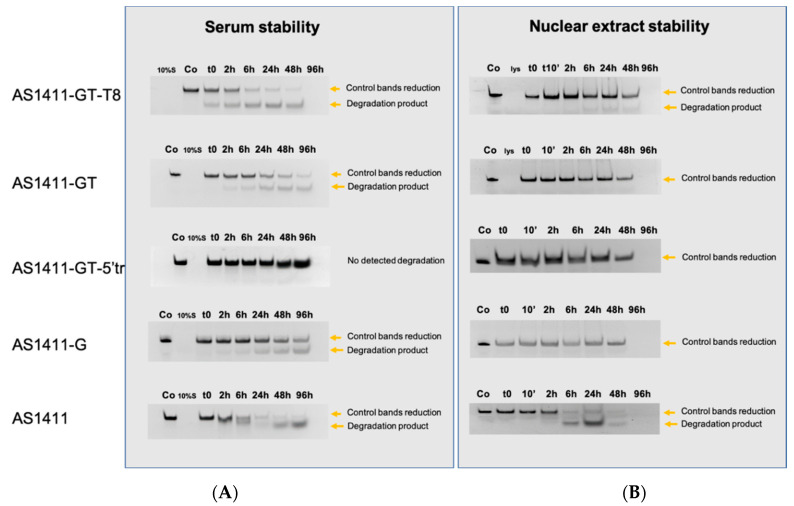
Stability of GROs in serum-containing medium and in nuclear extract. GROs were incubated with (**A**) 10% FBS or (**B**) 100 µg of nuclear extract at different times (0–96 h). Samples were analyzed by 16% polyacrylamide gel electrophoresis. Co = oligonucleotide alone, S = fetal bovine serum, lys = nuclear lysate.

Herein, we compared the nuclease resistance of the investigated GROs when placed in serum-containing medium (10% fetal bovine serum, FBS) and nuclear protein extracts, which simulate extracellular and intracellular conditions, respectively. For a highly sensitive detection, the GROs used in this experiment were labeled with 6-carboxyfluorescein (FAM) at 5′-end. The degradation profile of FAM-labelled GROs was analyzed at different times by polyacrylamide gel electrophoresis (PAGE). Some GROs showed the formation of fluorescent smear bands in the gel which suggests their progressive degradation from 3′-end, while others did not show smear bands, but only the disappearance of the band of the intact oligonucleotide at longer incubation times, which may be due to a very small amount of progressively degraded GROs (Figure 4). Interestingly, PAGE analysis revealed that AS1411-GT-5′tr, which adopts the most compact G4 structure and does not contain any central loop, has strong stability to serum nucleases. Indeed, the fluorescence of intact oligonucleotide is almost unchanged after 96 h of incubation (Figure 4A). Conversely, all the other GROs are significantly digested already after 6 h of incubation with serum nucleases. In particular, among them, AS1411-GT-T8, which contains the longer central loop, showed the weakest resistance to serum nucleases, as it is significantly degraded immediately after adding 10% FBS (Figure 4A), followed by AS1411-G and AS1411-GT that showed a progressive degradation after 2 h of incubation in the presence of 10% FBS.

Regarding the oligonucleotides’ stability against nuclear nucleases, all investigated GROs resulted to be completely digested upon 96 h of treatment, as confirmed by the disappearance of the fluorescent bands in the corresponding gels (Figure 4B). However, among them, AS1411-GT-5′tr, AS1411-G, and AS1411-GT showed the highest degree of resistance to these nucleases, since the fluorescent bands of the intact oligonucleotides are still visible up to 48 h of incubation with nuclear extract (Figure 4B). Conversely, AS1411 showed the weakest resistance to nuclear nucleases, as this GRO is significantly digested already after 6 h of incubation, followed by AS1411-GT-T8 which showed some degradation under the same conditions (Figure 4B).

Overall these results revealed different patterns of enzymatic degradation of GROs in the presence of serum or nuclear nucleases, indicating a different action of these enzymes, which is also a function of exposure time. In addition, the biological stability of GROs was found to depend on the length of the central loop, especially in the presence of serum nucleases. In fact, the long central loop seems to be a preferred site of hydrolysis for serum nucleases, while the absence of this loop seems to confer a high resistance to nuclease digestion to AS1411-GT-5′tr.

### 2.7. Evaluation of Oligonucleotide Stability to Serum by Means of FRET

**Figure 5:**
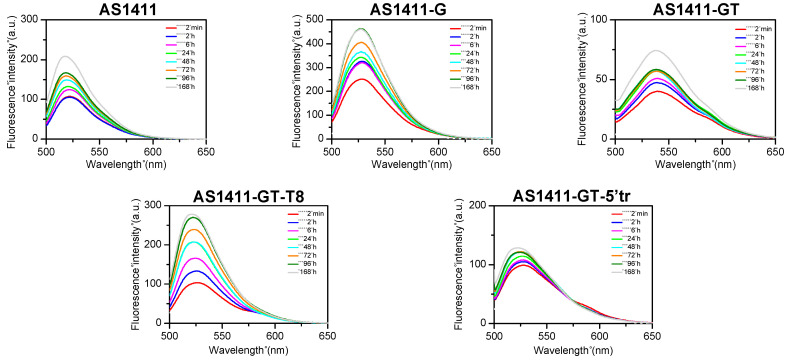
Time dependent fluorescence emission spectra of FAM/TAMRA-labelled GROs (0.1 μM) in the presence of 10% FBS (from 2 min to 168 h of incubation at 37 °C).

To further verify the stability of investigated GROs to serum nucleases, fluorescence resonance energy transfer (FRET) experiments were carried out. Dual fluorophore-labelled (FAM/TAMRA) oligonucleotides were prepared according to the principle of FRET [43,44]. These oligonucleotides are characterized by the presence of the donor fluorophore FAM (6-carboxyfluorescein) and the acceptor fluorophore TAMRA (6-carboxytetramethylrhodamine) that are covalently bound at 5′- and 3′-ends, respectively. Typically, when a G4 is folded, the two dyes are in close proximity so FAM fluorescence peak at 522 nm (upon excitation at 492 nm) is quenched by TAMRA [44,45]. On the other hand, if a G4 is unfolded or the oligonucleotide is degraded by nucleases, the distance-dependent interaction between the two dyes is disrupted, and an increase of the FAM fluorescence signal at 522 nm is observed [45]. Figure 5 shows the fluorescence spectra of the five FAM/TAMRA-labeled GROs in the presence of 10% FBS recorded at different times of incubation at 37 °C. For all GROs, an increase over time in the fluorescence signal at 522 nm was observed upon serum addition. However, the variation of fluorescence intensity over time occurred at a different extent for each GRO. In particular, it was lower in the case of AS1411-GT-5′tr, indicating its greater stability to the serum nucleases compared to the other GROs, in agreement with the results of the PAGE analysis reported above.

### 2.8. Evaluation of Antiproliferative Activity of GROs

**Figure 6:**
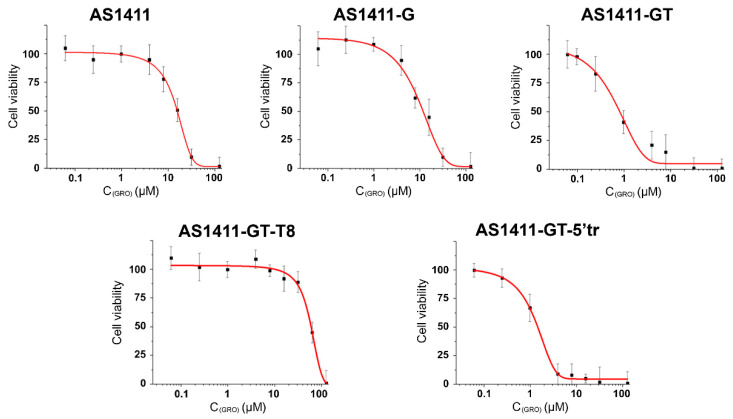
Viability (%) of MCF-7 cells treated with GROs at concentrations from 0.05 to 10 μM for 96 h.

To compare the antiproliferative activity of AS1411 with that of its derivatives against cancer cells, the colorimetric MTT assay was used. MCF-7 human breast adenocarcinoma cell line was chosen for these experiments as it was widely used to study GRO effects and, in particular, to evaluate the antiproliferative activity of AS1411 [4,7]. The results clearly show a dose-dependent cytotoxic effect on MCF-7 cells for all GROs (Figure 6). The most cytotoxic oligonucleotides of the series were AS1411-GT and AS1411-GT-5′tr (with an IC_50_ of 0.9 and 1.0 μM, respectively), which were found to be about 11–14 times more cytotoxic than AS1411 (IC_50_ = 13 μM) (Table 4). On the other hand, the antiproliferative effect of AS1411-G on MCF-7 cells (IC_50_ = 11 μM) was very similar to that of AS1411, while that of AS1411-GT-T8 was significantly weaker (IC_50_ = 53 μM). Interestingly, the antiproliferative activity of AS1411-GT and AS1411-GT-5′tr seems to be selective towards cancer cells, as the viability (%) of human dermal fibroblasts (HDF) treated with these GROs (at 1 and 10 μM doses) was significantly higher with respect to MCF-7 cells (Figure S4).

### 2.9. Induction of Apoptosis in MCF-7 Cells by GROs

Solid evidence has been provided that the interaction between AS1411 and nucleolin leads to the up-regulation of p53 and down-regulation of Bcl-2, both proteins responsible for cell apoptosis [46]. However, as described above, several nucleolin-independent mechanisms have been shown to contribute to the biological activity of AS1411 [10,13]. In this context, our results indicate that the GRO with lowest affinity for nucleolin, i.e., AS1411-GT-5′tr, actually turned out to be among the most cytotoxic GROs of the series. Therefore, we decided to explore its ability to induce cell apoptosis compared to AS1411. AS1411-GT was also added to this analysis since it showed a cytotoxic activity similar to AS1411-GT-5′tr and an affinity for nucleolin intermediate between those of AS1411 and AS1411-GT-5′tr.

**Figure 7:**
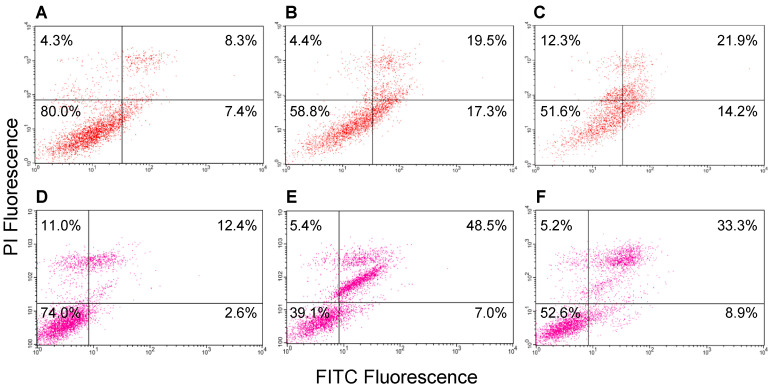
Apoptosis analysis with annexin V-FITC/PI double staining on MCF-7 cells treated for 96 h with (**A**,**D**) control, AS1411-GT-5′tr at (**B**) 1 μM and (**E**) 10 μM, and AS1411-GT at (**C**) 1 μM and (**F**) 10 μM. For all panels: upper left quadrants, necrotic cells; upper right, advanced apoptotic cells; lower left, viable cells; lower right, early apoptotic cells. These results are representative of three independent experiments.

**Figure 8:**
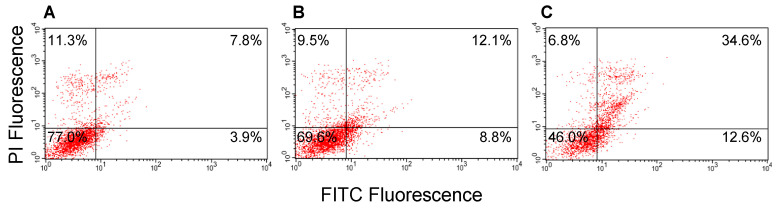
Apoptosis analysis with annexin V-FITC/PI double staining on MCF-7 cells treated for 96 h with (**A**) control, and AS1411 at (**B**) 1 μM and (**C**) 10 μM. For all panels: upper left quadrants, necrotic cells; upper right, advanced apoptotic cells; lower left, viable cells; lower right, early apoptotic cells. These results are representative of three independent experiments.

The analysis of the apoptotic profile of MCF-7 cells treated with the three GROs was performed by a double staining of cells with Annexin V and propidium iodine (PI), followed by analysis with flow cytometry. In particular, FITC-labeled Annexin V was used to specifically identify early and late apoptotic cells, while PI stains positively late-stage apoptotic and necrotic cells. Cells were first incubated for 96 h with 1 and 10 μM of the indicated GROs, followed by staining with PI and FITC Annexin V. The results showed in Figure 7 and Figure 8 reveal a similar behavior for the three GROs. Indeed, a highest population of cells in the apoptotic stage was observed in all three cases, although 10 μM AS1411-GT-5′tr treated cells exhibited a great percentage of cells in late apoptosis. In addition, the proportion of apoptotic cells increased in a dose-dependent manner (up to around 55% considering early and late apoptotic cells for AS1411-GT-5′tr) with the oligonucleotide concentration. Taken together, these results suggest once again that the nucleolin-mediated antiproliferative activity of GROs may be only one of the mechanisms by which GROs induce tumor cell death [47].

## 3. Discussion

G4-forming GROs demonstrated a great potential to be used in cancer therapy, being more specific against cancer cells and much less toxic for the organism than conventional methods of genotoxic chemotherapy. A general property of some GROs is the selective antiproliferative activity, which is mainly attributed to their nuclease resistance, efficient cancer cell uptake, and ability to bind and inhibit specific proteins involved in carcinogenesis and tumor progression [4], although it has been suggested that the cytotoxicity of guanine-based degradation products also contributes to their antiproliferative activity [14]. Investigations into the mechanism of AS1411 have revealed biological effects due to its interaction with nucleolin [9,10,46,48], as well as multiple nucleolin-independent effects [7,10].

Therefore, in this study, we designed and synthesized a small library of AS1411 derivatives with the aim to improve the anticancer properties of AS1411 and to elucidate which changes in the primary and secondary structure of this GRO are crucial for targeted interaction with cancer-related proteins and antiproliferative functions. As the parent oligonucleotide, three of the four derivatives (AS1411-G, AS1411-GT, and AS1411-GT-T8) had eight G-tracts, which in principle may allow the formation of two tandem G4s connected by a linker, but they differed from AS1411 in the number of Gs in the G-tracts and Ts in the loops, as well as in the central linker sequence and size (Table 1). In particular, the replacement of the ‘TTGT’ central linker with eight T residues was used to change the flexibility of structured GRO and to increase the distance between the two G4-forming motifs. Finally, a GRO consisting of the first nineteen residues at the 5′-end of AS1411-GT and AS1411-GT-T8 (i.e., AS1411-GT-5′tr), unable to form two tandem G4s, was also investigated.

NMR spectra of AS1411 and its derivatives definitely indicate the formation of G4s by these GROs. Broad overlapping proton signals of low intensity in the imino and aromatic regions are especially characteristic of the parent AS1411, which can be explained by the intrinsic structural polymorphism of AS1411 [11] and/or by the low thermal stability of two G-tetrad-layer G4 structure (s) formed by this oligonucleotide. Indeed, AS1411-GT-5′tr, capable of folding into a more stable G4 with three G-tetrad layers and having a lower potential for the formation of multiple G4s than AS1411, demonstrated more resolved imino signals with reduced overlap. Probably, the main reason for the heavily overlapping ^1^H imino resonances in the NMR spectra of our derivatives with repeated motifs was previously described in the study of d(GGGTGGGTGGGTGGGT) and related oligonucleotides [49], in which the authors attributed the low-resolved proton signals in the imino region to the quasi-symmetry of the structure arising from the repetitive nature of the sequence, which could hamper detailed structural analysis of G4s by NMR.

The major folding topologies and comparative thermodynamic stability of G4s adopted by GROs were evaluated by CD spectroscopy. It is now recognized that some G4 motifs can form structures of various architecture at different rates that could appear almost identical in thermodynamic, electrophoretic, and hydrodynamic properties, but exhibit different CD and NMR spectra, as well as kinetic stability [11,50]. As indicated by CD spectra, AS1411 and its derivatives fold into G4 structures with a parallel arrangement of G-tracts, both in K^+^ and PBS buffers. The parallel form is known to be predominant for G4 motifs with three single residue loops [31] and, generally, this conformation is more energetically favorable also for G4-forming motifs with three dinucleotide loops (as in AS1411-GT, AS1411-GT-T8, and AS1411-GT-5′tr) [31,51,52]. Significant dependence of *T*_1/2_ values on the heating rate for AS1411 and its longer derivatives in K^+^ buffer (Table 2) clearly indicate their non-equilibrium conformational transitions and sequential folding/unfolding processes that probably proceed through multistep pathways with intermediate states [53,54]. This can occur with both single G4s and, more likely, with systems containing multiple G4 units.

It was previously reported that the DNA sequences capable of forming two independent G4s separated by oligonucleotide linkers of various lengths can adopt a number of different coexisting folded states, many of them with roughly similar energies [11,55]. In some cases, structures with two tandem G4s behaving as independent beads-on-a-string were suggested to be the most favored [55,56,57], though models in which the tandem G4s stack on each other, interacting and forming higher-order structures, have also been proposed [58,59,60]. The structures obeying the model “beads-on-a-string” unfold at lower melting temperatures than individual G4s [55,61], while the higher-order G4 ensembles should be more stable than the individual G4 units reflecting stabilizing interactions at the G4-G4 interface [54,62]. It is interesting to note that only parallel-stranded G4s are known to be prone to inter-G4 π–π stacking interactions [29,54,63,64].

According to our CD melting data, GROs with two contiguous G4-forming motifs unfold at significantly higher temperatures than AS1411-GT-5′tr containing a single G4 motif with a similar sequence. For this reason, we assume that these GROs may predominantly form higher-order ensembles stabilized by stacking interactions between outer G-tetrads from adjacent G4 units. The comparative analysis of the thermodynamic stability of GROs under identical folding and melting conditions (Table 2) showed that AS1411 (having only two Gs for each G-tract) is the less stable of the series. Among the GROs with three G residues for each G-tract, AS1411-G (having six single-thymine loops) has shown to be the most stable (*T*_1/2_ > 93 °C in K^+^ buffer). It was shown that independently on the orientation of DNA strands, the thermodynamic stability of G4s decreases as the length of loops increases [16,51,65]. Indeed, the *T*_1/2_ values of AS1411-GT are significantly lower than AS1411-G due to the additional T residue for each of the six single-nucleotide loops. On the other hand, an increase in *T*_1/2_ values was observed for AS1411-G, AS1411-GT, and AS1411-GT-T8 as their concentration increased from 1 to 20 µM. This suggests the possible presence of a certain amount of dimeric forms. Since the dependence of the thermal stability on DNA concentration is significantly higher for AS1411-GT-T8 than AS1411-G and AS1411-GT, it can be speculated that the presence of eight T residues central linker may increase the percentage of bimolecular structures.

A comparison of the binding affinities of nucleolin to AS1411 and its derivatives revealed the following affinity ranking: AS1411-GT-T8 >> AS1411 > AS1411-G > AS1411-GT >> AS1411-GT-5′tr, and equilibrium dissociation constants (*K*_D_) in the range 12–2800 nM (Table 3). Therefore, binding of nucleolin to AS1411-GT-T8 was found to be more than 200-fold stronger than to AS1411-GT-5′tr. These findings are in agreement with the literature data unambiguously showing that nucleolin discriminates drastically between long-loop and short-loop G4s [17,18]. Moreover, an inspection of the kinetics data reveals valuable information on the association and dissociation rates for GRO/protein complex formation. Indeed, the comparison of the kinetic constants reveals that the *k*_on_ value obtained for AS1411 (2.9 × 10^3^ M^−1^·s^−1^) is not so different from those obtained for AS1411-GT-T8 and AS1411-GT-5′tr (3.5 × 10^3^ and 0.75 × 10^3^ M^−1^·s^−1^, respectively), while larger differences are found for the *k*_off_ values. In particular, the *k*_off_ values observed for AS1411-GT-5′tr (21.0 × 10^−4^ s^−1^) and AS1411-GT-T8 (0.41 × 10^−4^ s^−1^) are about 8.4-fold greater and 6.3-fold lower than for AS1411 (2.5 × 10^−4^ s^−1^), respectively, thus suggesting that the large differences in affinity arise from the dissociation rates. This may be due to the presence and the different length and flexibility of the central linker which modulates the conformational flexibility of GROs and, consequently, their kinetics and thermodynamics of binding to nucleolin.

On the other hand, the susceptibility of the central loop to the action of nucleases was shown in experiments in which GROs’ resistance to cleavage was evaluated in serum-containing medium and in nuclear extract. As seen from oligonucleotide degradation patterns, only AS1411-GT-5′tr lacking the central linker was stable in these conditions. Therefore, the assumption that the antiproliferative activity of GROs may also be contributed by the cytotoxicity of their guanine-based degradation products (i.e., deoxyguanosine monophosphate, deoxyguanosine, and guanine) does not emerge from this study. Indeed, AS1411-GT-5′tr, which showed the best cytotoxic effect (together with AS1411-GT) on MCF-7 cells of all the GROs studied (Table 4), turned out to be the most resistant to nuclease degradation. This is in agreement with the vast majority of published data indicating that only those GROs that are folded into stable G4 structures exhibit pronounced biological activity [6,7,66,67]. In addition, the hypothesis that GROs’ cytotoxic activity is not simply due to their interaction with nucleolin but to multi-targeted effects, including the targeting of other G4-recognizing proteins such as TOP1 [40,41], is once again supported by the data reported in this study. Indeed, all GROs studied here exhibited a significant inhibitory effect against TOP1, but their ability to inhibit TOP1 does not fully reflect their antiproliferative activity on MCF-7 cells (Table 4).

In summary, we have investigated a small library of AS1411 derivatives differing in the number of Gs in the G-tracts as well as in the sequence and size of the loops. Some of these derivatives showed improved antiproliferative activity than the parent GRO. More importantly, our results support the hypothesis that there is no single mechanism of action explaining the cytotoxic activity of GROs, which does not appear to be simply related to the targeting of nucleolin or TOP1, but likely to multi-targeted effects. Therefore, important questions still need to be answered to optimize the use of GROs as cancer-targeting agents. Further research to elucidate the molecular mechanisms involved would be of benefit and may lead to important discoveries in cancer cell biology and to novel strategies for targeting cancer cells.

## 4. Materials and Methods

### 4.1. Materials

The Human Recombinant Nucleolin Protein (NM_005381) was purchased from OriGene Technologies GmbH (Herford, Germany). Phosphoramidite and controlled pore glass supports for DNA synthesis were purchased from Link Technologies (Bellshill, UK). Sensor chips, amino coupling reagents, and buffers for surface plasmon resonance (SPR) measurements were purchased from GE Healthcare Europe GmbH (Milan, Italy). All common chemicals, reagents, and solvents were purchased from Merck KGaA (Darmstadt, Germany) unless otherwise stated.

### 4.2. Oligonucleotide Synthesis and Sample Preparation

All DNA oligonucleotides were chemically synthesized on an ABI 394 DNA/RNA synthesizer (Applied Biosystem) at 5 or 1 μmol scale, using the standard β-cyanoethylphosphoramidite solid-phase chemistry as described elsewhere [68]. After synthesis, the oligonucleotides were detached from the support and deprotected by treating with concentrated aqueous ammonia at 55 °C for 12 h. Filtrates and washings were concentrated under reduced pressure, dissolved in water, and purified by HPLC using standard protocols. Isolated oligonucleotides have been shown to be more than 98% pure by NMR. The oligonucleotide concentration was determined spectrophotometrically by UV adsorption measurements at 90 °C using molar extinction coefficients calculated at 260 nm by the nearest-neighbor model [69]. G4s were prepared in two different phosphate-buffered solutions (pH 7.4): 10 mM Li_3_PO_4_, 10 mM KCl, 0.2 mM EDTA (K^+^ buffer), and 10 mM Na_2_HPO_4_, 1.8 mM KH_2_PO_4_, 137 mM NaCl, 2.7 mM KCl (PBS buffer). Samples were then heated at 90 °C for 5 min, gradually cooled to room temperature overnight, and finally incubated at 4 °C for 24 h, before data acquisition.

### 4.3. NMR Measurements

NMR experiments were performed on a 700 MHz Varian Unity INOVA spectrometer equipped with a cryoprobe. One dimensional ^1^H NMR spectra of the samples in K^+^ or PBS buffer solutions were recorded at 25 °C using pulsed-field gradient DPFGSE for H_2_O suppression [70]. Data were processed on iMAC running iNMR software (www.inmr.net). DNA samples were prepared at 0.2–0.4 mM strand concentration in 0.6 mL of H_2_O/D_2_O (9:1) buffer solution. The samples were heated at 95 °C for 5 min, then slowly cooled to room temperature overnight to achieve the correct folding of the oligonucleotides, and finally incubated at 4 °C for 24 h, before data acquisition.

### 4.4. Circular Dichroism Experiments

CD experiments were carried out on a Jasco J-815 spectropolarimeter equipped with a Peltier cell holder for temperature control (PTC-423S/15). All the experiments were carried out both in K^+^ and PBS buffers, using GRO concentrations of 1 and 20 μM. Spectra were recorded at 20 °C in a wavelength range of 220–360 nm, using 1 or 10 mm path length quartz cuvettes, 1 nm bandwidth, 1 s response time, and a scan rate of 100 nm/min. Spectra were averaged over three scans and buffer baseline was subtracted from each spectrum. CD melting experiments were performed at 0.5 and 2 °C/min heating rates by following the CD signal at 263 nm, which corresponds to the wavelength of the maximum intensity value of the respective spectra. Melting temperatures were determined from curve fitting using Origin 7.0 (OriginLab, Northampton, MA, USA).

### 4.5. Surface Plasmon Resonance Experiments

SPR experiments were performed on a Biacore X100 instrument (GE Healthcare). Nucleolin protein was immobilized on the surface of a CM5 sensor chip using a standard Amine Coupling Kit and HBS-EP buffer (HEPES 10 mM, NaCl 150 mM, EDTA 3 mM, 0.005% surfactant P20, pH 7.4). The flow cell surfaces were activated by a 1:1 mixture of 0.1 M NHS (N-hydroxysuccinimide) and 0.1 M EDC (3-(N,N-dimethylamino)propyl-N-ethylcarbodiimide) at a flow rate of 10 μL/min. Afterward, the protein (25 μg/mL in 10 mM sodium acetate, pH 4.5) was immobilized on the sample flow cell, leaving the reference cell as blank. After immobilization, the remaining reactive sites were blocked by injection of 1.0 M ethanolamine at 10 μL/min over the chip surface. GRO samples were injected at different concentrations (from 2 to 32 μM), using PBS as running buffer. All interactions were performed at 25 °C and at the flow rate of 30 μL/min, with association times of 60 s. The sensor surface was regenerated by using 5 mM NaOH for 12 s. Curves obtained on the reference surface were subtracted from the curves recorded on the protein-functionalized one, allowing the elimination of refractive index changes due to buffer effects. Data were fit to a single cycle kinetic interaction model, using the global data analysis option available within the BIAevaluation software (GE Healthcare, Uppsala, Sweden) provided with the instrument. Standard errors for the constants were calculated from the statistics on the data fit and presented as percentage values.

### 4.6. Assessment of the Level of Inhibition of Topoisomerase I

To determine the ability of GROs to inhibit the activity of topoisomerase I (TOP1) in vitro, one activity unit of catalytically active TOP1 enzyme (TopoGen, Port Orange, FL, USA) was incubated with 0.2 μg pUC19 supercoiled plasmid DNA in a commercial buffer solution, with various amounts of the investigated oligonucleotides. The reaction was performed at 37 °C for 30 min and stopped by adding SDS to a final concentration of 1% and treating with proteinase K (50 μg/mL) for 60 min at 55 °C. Reaction products were separated by electrophoresis in 1% agarose gels using the TAE buffer. A high-density buffer solution (30% glycerol, 0.25% bromophenol blue) was used to load the plasmid DNA into the agarose gel wells. Electrophoresis was performed using a maximum electric field voltage of 2 V/cm. Gels were stained with an aqueous solution of ethidium bromide (0.5 μg/mL) and visualized by using a UV light source (240–360 nm) to excite the fluorescent DNA molecules. The resulting images were processed using ImageJ. For each concentration of GRO, the degree of enzymatic inhibition (*I*) was calculated by using the following equation:

(1) $$I=\frac{S-S_{0}}{S_{control}-S_{0}}\times 100$$

where *S* is the amount of supercoiled plasmid after treatment with TOP1 in the presence of GRO, *S*_0_ is the amount of supercoiled plasmid in a sample containing no oligonucleotide, and *S_control_* is the amount of the supercoiled plasmid in a control sample without TOP1. After that, the degree of inhibition was plotted against the logarithm of GRO concentration, and the half-maximal inhibitory concentration (IC_50_) was determined from curve fitting. Each measurement was carried out in triplicate.

### 4.7. Serum Degradation Assay

The degradation profile of GROs upon addition of serum was analyzed at different times by PAGE and FRET analysis. For a highly sensitive detection on PAGE, GROs used in this experiment were labeled with 6-carboxyfluorescein (FAM) at 5′ end. Oligonucleotides (0.5 μg) were incubated for different times with 10 μL DMEM supplemented with 10% FBS. The reactions were stopped by adding 0.1 mM EDTA and incubating the mixture at 100 °C for 5 min. The reaction products were analyzed by 16% polyacrylamide gel electrophoresis (PAGE). Prior to loading the mixtures onto the gel, 3 μL of glycerol solution (30% *v*/*v*) was added. Gels were run at 100 V for 45 min in Tris-Glycine buffer (pH 8.3). Gels were imaged by the UV transilluminator of a ChemiDoc XRS System (Bio-Rad Laboratories) and analyzed with the QuantityONE software. As far as FRET experiments are concerned, FAM/TAMRA-labelled oligonucleotides (0.1 μM) were incubated in PBS at 37 °C in the presence of 10% FBS. Fluorescence spectra were recorded at different times of incubation on a FP-8300 spectrofluorometer (Jasco) equipped with a Peltier temperature controller accessory (Jasco PCT-818). A sealed quartz cuvette with a path length of 1 cm (2 mL) was used. Measurements were made with excitation at 492 nm and emission from 500 to 650 nm at 100 nm/min scan rate, with both excitation and emission slits set at 5 nm.

### 4.8. Cell line and Culture Conditions

Human breast adenocarcinoma (MCF-7) cell line (ATCC) was grown in DMEM supplemented with 10% FBS, 2.5 mM glutamine, 100 U/mL penicillin, and 100 µg/mL streptomycin (Euroclone). Normal human dermal fibroblasts (HDF), kindly provided by Dr. Annalisa Tito (Arterra Bioscience), were grown in DMEM supplemented with 10% FBS, 1% glutamine, 100 U/mL penicillin, and 100 µg/mL streptomycin [71]. Cells were maintained in humidified air containing 5% CO_2_ at 37 °C.

### 4.9. Nuclear Protein Extraction

A confluent plate of MCF-7 cells was detached with trypsin-EDTA (Euroclone) in order to detach the cells, which were collected, pelleted, and washed in ice-cold PBS 1X. The nuclear extract was obtained following a protocol described in Luo et al. [72]. In brief, after washing, the cellular pellet was resuspended in a volume of hypo-osmotic lysis buffer (0.3 M sucrose, 2% Tween 40, 10 mM HEPES-KOH pH 7.9, 10 mM KCl, 1.5 mM MgCl_2_, 0.1 mM EDTA and protease inhibitors) corresponding to 5 times the packed cell volume. Successively, cells were pipetted 100 times using a micropipette with a 200 μL pipette tip in order to homogenize the sample. Enucleated samples were overlaid on 1 mL of 1.5 M sucrose buffer (1.5 M sucrose, 10 mM HEPES-KOH pH 7.9, 10 mM KCl, 1.5 mM MgCl_2_, 0.1 mM EDTA, and protease inhibitors) and centrifuged for 10 min. The nuclear pellets were washed in 1 mL of low-salt wash buffer (10 mM HEPES-KOH pH 7.9, 10 mM KCl, 1.5 mM MgCl_2_, 0.1 mM EDTA and protease inhibitors), and pelleted again. The isolated nuclear pellets were resuspended in 50 μL of high-salt extraction buffer (20 mM HEPES-KOH pH 7.9, 420 mM NaCl, 1.5 mM MgCl_2_, 0.2 mM EDTA, 25% glycerol and protease inhibitors), and placed on ice for 20 min. Then, the samples were centrifuged for 20 min and the supernatants collected. Protein concentration was estimated by Bradford reagent.

### 4.10. Evaluation of GRO’s Degradation by Nuclear Extract

FAM-labeled GROs (0.5 μg in 10 μL PBS) were incubated at 37 °C with 13 µg of nuclear extract for different time points. Reactions were stopped by adding 0.1 mM EDTA, followed by heating at 100 °C for 5 min. The reaction products were analyzed by 16% polyacrylamide gel electrophoresis (PAGE) as described above (see Section 4.7).

### 4.11. Cell Viability Assays

The cytotoxic effect of GROs was evaluated using the MTT assay. For the analysis, MCF-7 and HDF cells were planted in 96-well plates with 1000 cells in each well (cell medium volume—200 μL). Cells were incubated for 24 h at 37 °C, after which GROs were added to the culture medium in a volume of 10 μL with the concentration necessary to achieve the desired concentration of DNA in the medium. MCF-7 cells were then incubated for 96 h at 37 °C, 10 μL of MTT solution (5 mg/mL MTT, 0.9 M NaCl) was added to each well and incubated for 4 h at 37 °C. The culture medium was removed and 100 μL DMSO was added to dissolve the precipitated formazan. The optical density was measured at 570 nm on a Multiscan FC spectrophotometer (ThermoScientific). MCF-7 cell viability at each concentration of GRO was calculated by the equation:

(2) $$Cell\;viability=\frac{OD-OD_{0}}{OD_{control}-OD_{0}}\times 100$$

where *OD* is the optical density of the sample, *OD*_0_ is the optical density of the background signal, and *OD_control_* is the optical density of the control sample (without the addition of a GRO). Each sample was measured in triplicate. For each GRO, the dependence of MCF-7 cell survival on the concentration was determined from which the half-maximum concentration of inhibition (IC_50_) was determined, i.e., the concentration of the GRO at which a 50% decrease in the number of cells was observed compared to the control sample. HDF cell viability was determined after 78 h of treatment with 1 and 10 µM of each GRO. The plates were analyzed by using an EnSpire microplate reader (Perkin Elmer) at 570 nm. The mean value ±SE of the adherent cells for each treatment was expressed as the percentage of the cell number with respect to the untreated cells (control). Statistical differences were determined by the Student’s test, paired, two-sided. Experiments were performed in triplicate and repeated at least three times; a *p* value < 0.05 was considered significant.

### 4.12. Apoptosis Experiments

MCF-7 were seeded at 3000 cells/cm^2^ in a six-well plate and treated at 37 °C for 96 h with GROs at 1 or 10 μM concentration. The assay was performed using the FITC annexin-V Apoptosis Detection kit (Pharmingen BD) as previously described [71,73]. Briefly, after incubation, the untreated and treated cells were trypsinized, washed twice with PBS, and resuspended at a concentration of 1 × 10^6^ cells/mL in the binding buffer, according to manufacturer’s instructions. Next, 5 μL of FITC-Annexin-V and 5 μL of propidium iodine were added to cell suspensions (100 μL) and incubated for 15 min at 25 °C in the dark. The percentage of cells undergoing apoptosis or necrosis was quantified using a flow cytometer equipped with a 488 nm argon laser (Becton-Dickinson) by Cell Quest software [71,73]. All FACS analyses were performed at least 3 times.

## Figures and Tables

**Table 1:** List of the investigated G-quadruplex-forming guanine-rich oligonucleotides (GROs).

Name	Sequence (5′–3′) ^1^
AS1411	GGTGGTGGTGG*TTGT*GGTGGTGGTGG
AS1411-G	GGGTGGGTGGGTGGG*TTGT*GGGTGGGTGGGTGGG
AS1411-GT	GGGTTGGGTTGGGTTGGG*TTGT*GGGTTGGGTTGGGTTGGG
AS1411-GT-T8	GGGTTGGGTTGGGTTGGG*TTTTTTTT*GGGTTGGGTTGGGTTGGG
AS1411-GT-5′tr	GGGTTGGGTTGGGTTGGGT

^1^ G-runs are underlined, residues belonging to the central linker are in italic.

**Table 2:** Apparent melting temperatures (*T*_1/2_) of GROs in K^+^ and PBS buffers at different DNA concentration and heating rate determined by circular dichroism (CD) melting curves.

	*T*_1/2_ (°C) ^1^ in K^+^ Buffer				*T*_1/2_ (°C) ^1^ in PBS Buffer			
	1 µM		20 µM		1 µM		20 µM	
	0.5 °C/min	2 °C/min	0.5 °C/min	2 °C/min	0.5 °C/min	2 °C/min	0.5 °C/min	2 °C/min
AS1411	48	54	50	55	38	39	41	44
AS1411-G	n.d.	n.d.	n.d.	n.d.	86	86	93	93
AS1411-GT	72	79	76	85	60	60	64	66
AS1411-GT-T8	70	72	92	93	65	67	88	90
AS1411-GT-5′tr	59	60	59	61	51	52	51	54

^1^ Errors were ± 1 °C. n.d. = not determinable (*T*_1/2_ > 93 °C).

**Table 3:** Kinetic parameters and dissociation constants for the interaction of GROs with nucleolin determined by SPR.

Analyte	*k*_on_ × 10^3^ (M^−1^ · s^−1^) ^1^	*k*_off_ × 10^−4^ (s^−1^) ^1^	*K*_D_ (nM) ^2^
AS1411	2.90	2.50	86
AS1411-G	4.60	7.90	170
AS1411-GT	0.47	2.40	510
AS1411-GT-T8	3.50	0.41	12
AS1411-GT-5′tr	0.75	21.00	2800

^1^ Errors were within 5%. ^2^ Errors were within 10%.

**Table 4:** Biological activity of GROs.

GRO	TOP1 Inhibition	Antiproliferative Activity on MCF-7 Cells (96 h)
	IC_50_ (μM)	IC_50_ (μM)
AS1411	4.8 ± 0.5	13 ± 2
AS1411-G	2.1 ± 0.4	11 ± 3
AS1411-GT	0.71 ± 0.08	0.9 ± 0.3
AS1411-GT-T8	0.20 ± 0.08	53 ± 8
AS1411-GT-5′tr	1.2 ± 0.2	1.0 ± 0.2

## Supplementary Materials

Supplementary materials can be found at https://www.mdpi.com/1422-0067/21/20/7781/s1.

## Abbreviations

Bcl-2	B-cell lymphoma 2
CD	circular dichroism
CPG	controlled-pore-glass
DMEM	Dulbecco’s modified Eagle’s medium
DMSO	dimethyl sulfoxide
DPFGSE	double pulsed field gradient spin echo
FACS	fluorescence-activated cell sorting
FAM	6-carboxyfluorescein
FBS	fetal bovine serum
FITC	fluorescein isothiocyanate
FRET	fluorescence resonance energy transfer
G4	G-quadruplex
GRO	guanine-rich oligonucleotide
HDF	human dermal fibroblasts
HPLC	high-performance liquid chromatography
NMR	nuclear magnetic resonance
PAGE	polyacrylamide gel electrophoresis
SCK	single cycle kinetics
SPR	surface plasmon resonance
STAT3	signal transducer and activator of transcription 3
TAE	tris-acetate-EDTA
TAMRA	6-carboxytetramethylrhodamine
TOP1	topoisomerase I
