# Supplementary material for: Toward G-Quadruplex-Based Anticancer Agents: Biophysical and Biological Studies of Novel AS1411 Derivatives
Source: Int J Mol Sci. 2020 Oct 21;21(20):7781. doi: 10.3390/ijms21207781 (PMC7590035; doi:10.3390/ijms21207781)
Supplement: Supplementary file 1 [file ijms-21-07781-s001.pdf]

Supplementary Material

Toward G-Quadruplex-Based Anticancer Agents: Biophysical and Biological Studies of Novel AS1411 Derivatives

Anna M. Ogloblina [1,2], Nunzia Iaccarino [2], Domenica Capasso [3], Sonia Di Gaetano [4], Emanuele U. Garzarella [2], Nina G. Dolinnaya [5], Marianna G. Yakubovskaya [1], Bruno Pagano [2,*], Jussara Amato [2,*] and Antonio Randazzo [2]

1 N.N. Blokhin National Medical Research Center of Oncology, Ministry of Health, 115478 Moscow, Russia; ann.ogloblina@gmail.com (A.M.O.); mgyakubovskaya@mail.ru (M.G.Y.)

2 Department of Pharmacy, University of Naples Federico II, Via D. Montesano 49, 80131 Naples, Italy; nunzia.iaccarino@unina.it (N.I.); emanueleugo.garzarella@unina.it (E.U.G.); antonio.randazzo@unina.it (A.R.)

3 Center for Life Sciences and Technologies (CESTEV), University of Naples Federico II, Via A. De Amicis 95, 80145 Naples, Italy; domenica.capasso@unina.it

4 Institute of Biostructures and Bioimaging, National Research Council, Via Mezzocannone 16, 80134 Naples, Italy; digaetan@unina.it

5 Department of Chemistry, Lomonosov Moscow State University, 119991 Moscow, Russia; dolinnaya@hotmail.com

* Correspondence: bruno.pagano@unina.it (B.P.); jussara.amato@unina.it (J.A.); Tel.: +39-081678641 (B.P.); +39-081678630 (J.A.)

Table of contents **Page**

Table of contents	Page
Figure S1	S2
Figure S2	S3
Figure S3	S4
Figure S4	S4

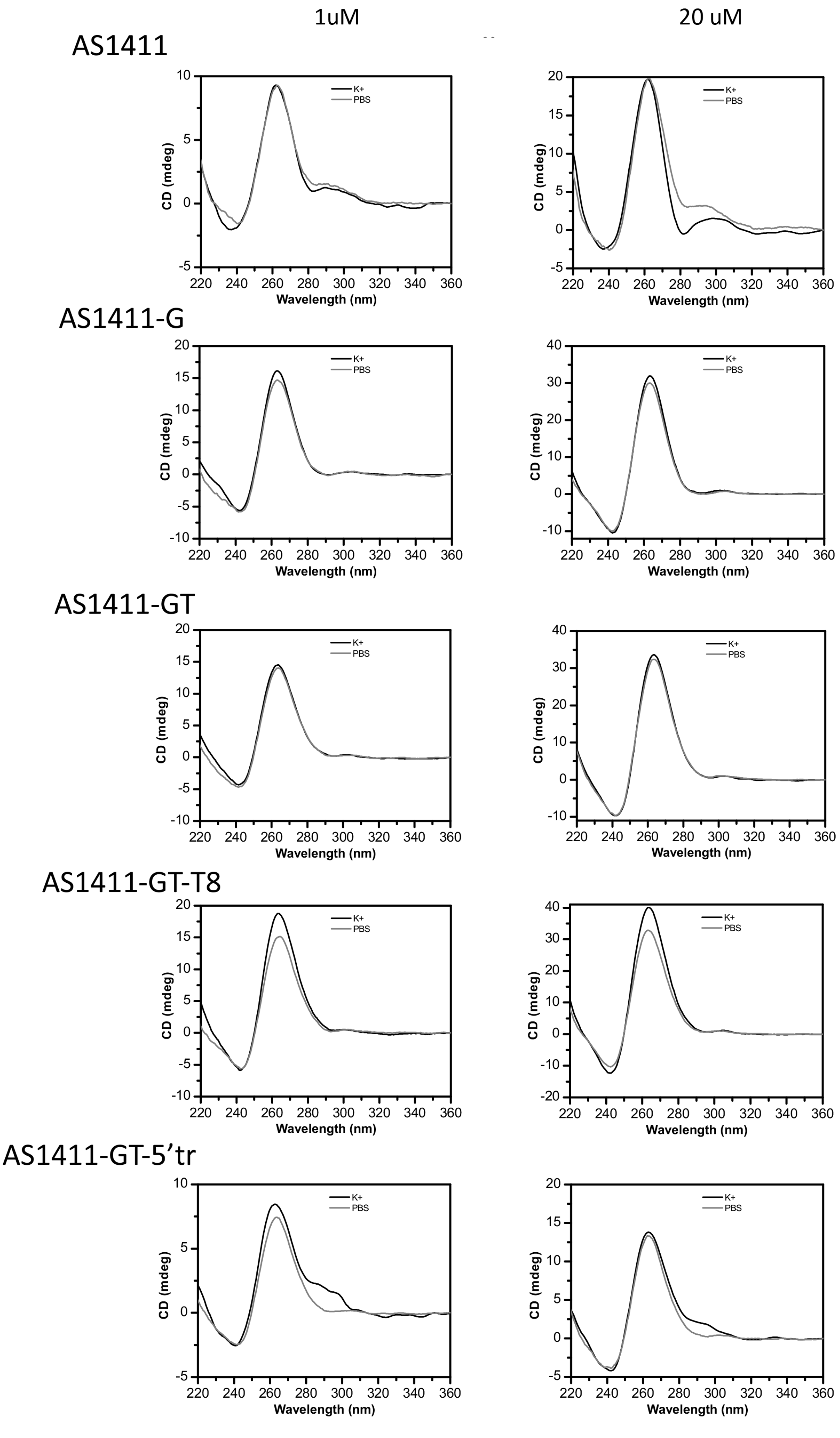

Figure S1. CD spectra of investigated oligonucleotides recorded at 20 °C.

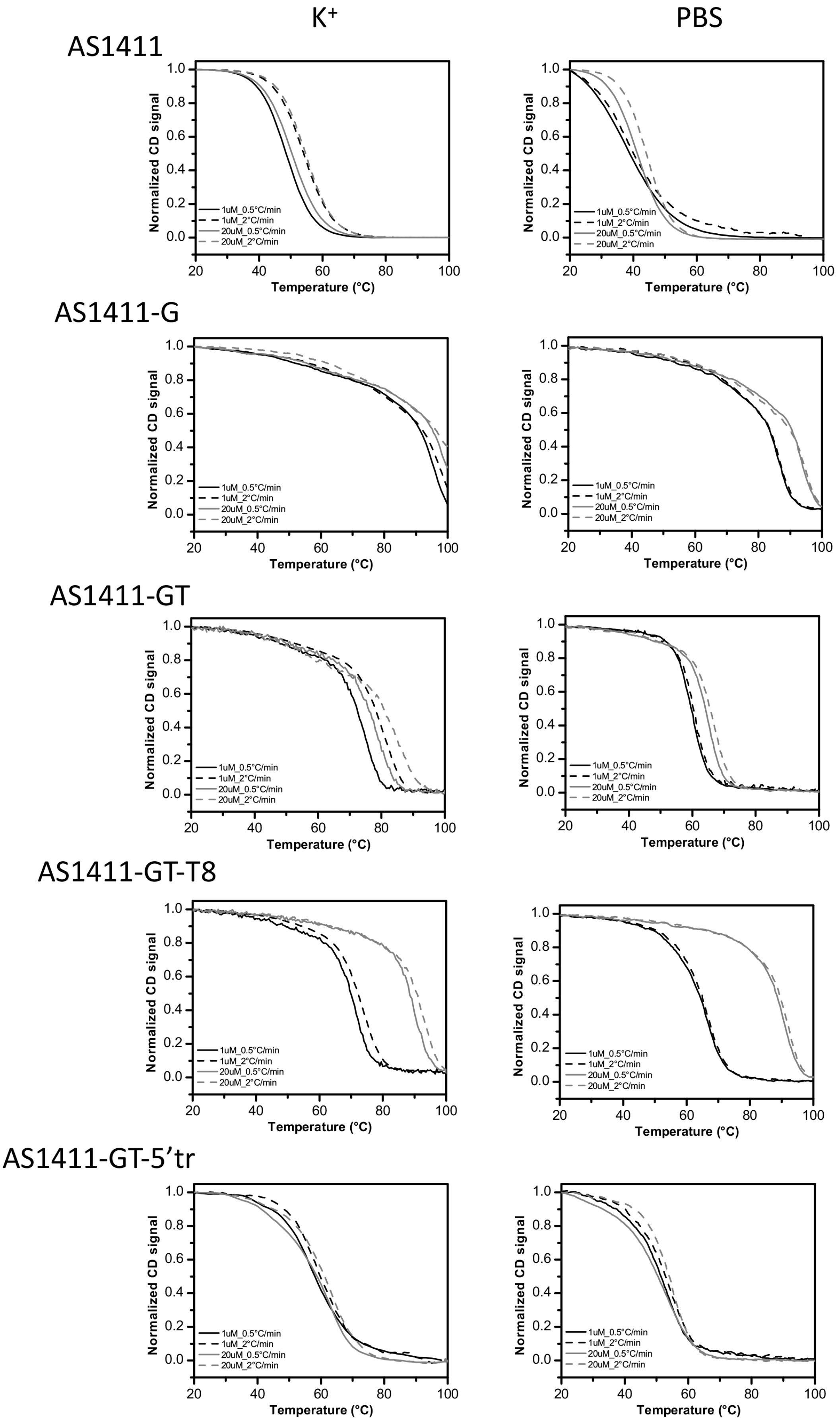

Figure S2. CD melting curves of investigated oligonucleotides recorded by following the CD signal at 263 nm.

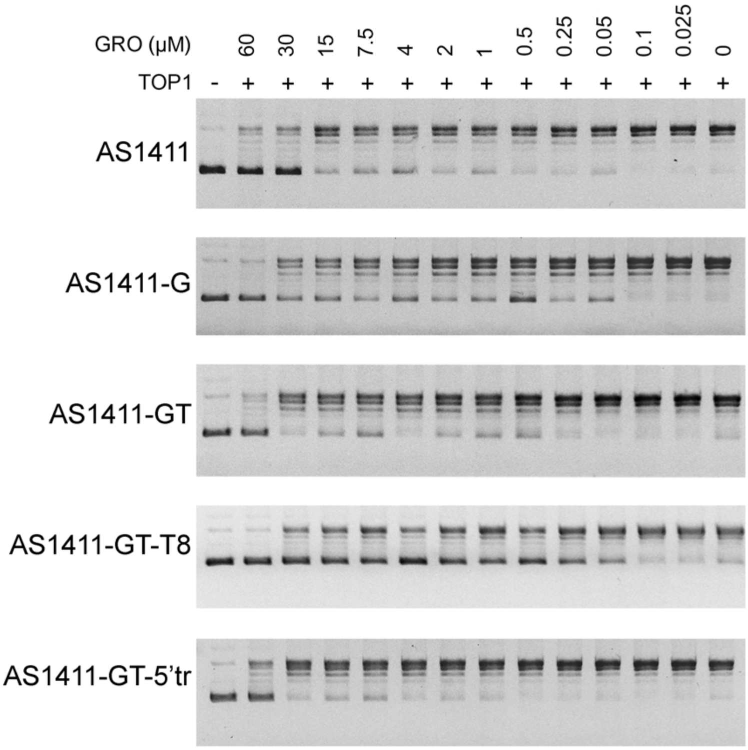

Figure S3. TOP1-catalyzed relaxation of supercoiled pUC19 plasmid in the presence of GROs. Topoisomers were separated in 1% agarose gel. The concentration of each GRO (μM) is indicated above the corresponding lane. The first lane on the left corresponds to the reaction mixture containing no enzyme. For visualization, gels were stained with ethidium bromide.

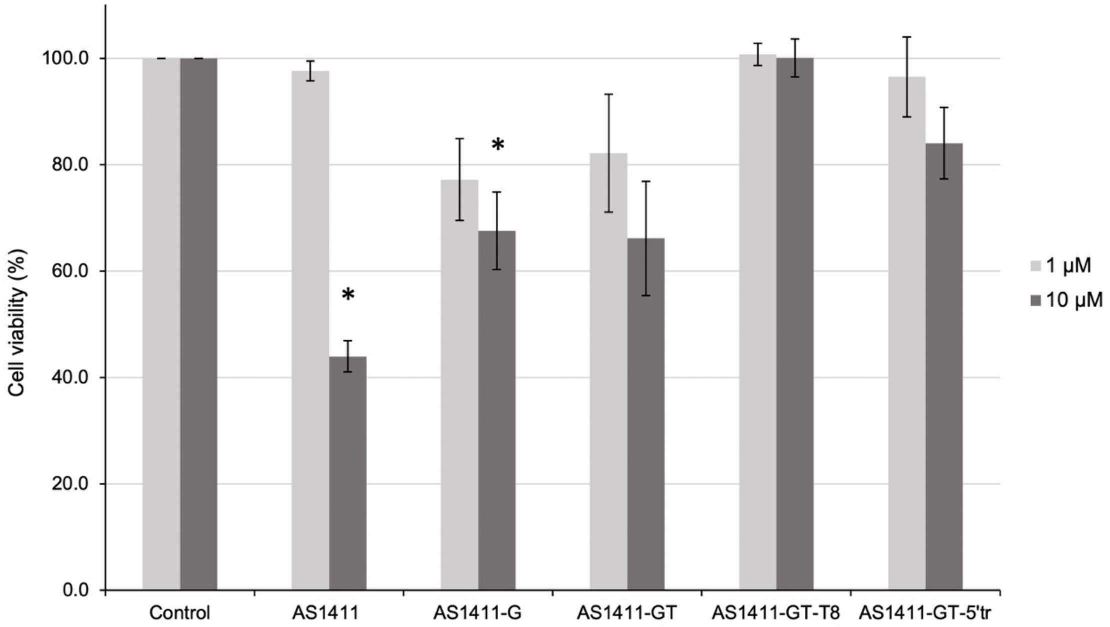

Figure S4. Effect of GROs on normal HDF cell proliferation. The cells were incubated at 37 °C in the presence of GROs at 1 or 10 μM concentration for 78 h. The results are presented as the percentage of viable cells with respect to the control (untreated cells) and are expressed as means ± SE of at least three independent experiments performed in triplicate, * $p < 0.05$.
